# EHMTI-0104. VIP levels in peripheral blood outside migraine attacks as a potential biomarker of cranial parasympathetic activation in chronic migraine

**DOI:** 10.1186/1129-2377-15-S1-F21

**Published:** 2014-09-18

**Authors:** E Cernuda-Morollón, P Martínez-Camblor, E Serrano-Pertierra, C Ramón, D Larrosa, J Pascual

**Affiliations:** 1 Neurology, Hospital Universitario Central de Asturias and Ineuropa, Oviedo, Spain; 2 OIB, Oficina Investigación Sanitaria, Oviedo, Spain

## Aim

To determine vasoactive intestinal peptide (VIP) levels outside migraine attacks in peripheral blood as a potential biomarker for chronic migraine (CM).

## Methods

Women older than 17 and diagnosed as CM were recruited. Matched women with no headache history and with episodic migraine (EM) served as controls, together with a series of patients with cluster headache in a pain-free period. VIP levels were determined in samples obtained from the antecubital vein by ELISA outside a migraine attack and having taken no symptomatic medication. Due to ethical reasons, preventatives were not stopped.

## Results

We assessed plasma samples from 119 women with CM, 33 healthy women, 51 matched women with EM and 18 patients (16 males) with cluster headache matched for age. VIP levels were significantly increased in CM (165.1 pg/ml) as compared to control healthy women (88.5 pg/ml) and episodic cluster headache patients (101.1 pg/ml). VIP levels in EM (134.9 pg/ml) were significantly higher as compared to controls and numerically lower that those of CM. Thresholds of 71.8 and 164.5 pg/ml optimize the sensitivity and specificity to differentiate CM from healthy controls and EM, respectively. Variables such as age, CM duration, the presence of aura, analgesic overuse, depression, fibromyalgia, vascular risk factors, history of triptan consumption or kind of preventative treatment did not influence VIP levels.

## Conclusion

Increased interictal VIP level measured in peripheral blood could be a biomarker helping in CM diagnosis, though it does not clearly differentiate between EM and CM. Supported by PI11/00889 FISSS and Allergan-Eurasia MAT/ISS/NS/CM/003 grants

